# Supplementary material for: The interactome of intact mitochondria by cross-linking mass spectrometry provides evidence for coexisting respiratory supercomplexes
Source: Mol Cell Proteomics. 2017 Dec 8;17(2):216–32. doi: 10.1074/mcp.RA117.000470 (PMC5795388; doi:10.1074/mcp.RA117.000470)
Supplement: Supplemental Data [file supp_RA117.000470_133922_0_supp_23978_fzffwf.zip › spectra_annotation/mito_spectra_annotation/164-1-3-1-12-1.pdf]

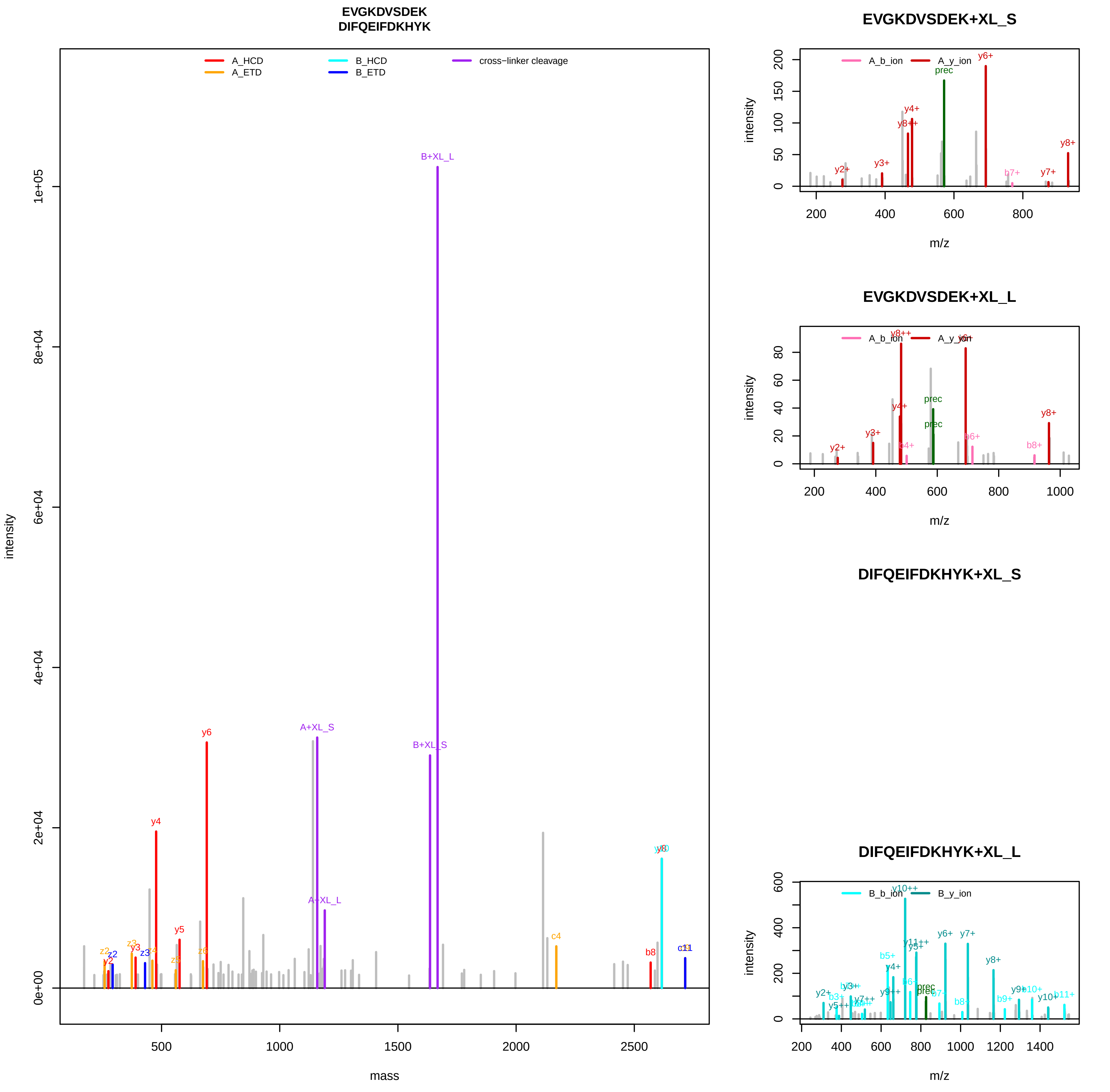
EVGKDVSDEK
DIFQEIFDKHYK
A_HCD
A_ETD
B_HCD
B_ETD
cross−linker cleavage
B+XL_L
A+XL_S
B+XL_S
A+XL_L
y2
y3
y4
y5
y6
b8
y8
z2
z3
z5
z6
c4
c11
intensity
mass
EVGKDVSDEK+XL_S
A_b_ion
A_y_ion
prec
y2+
y3+
y4+
y8++
y6+
b7+
y7+
y8+
m/z
EVGKDVSDEK+XL_L
b4+
b6+
b8+
DIFQEIFDKHYK+XL_S
DIFQEIFDKHYK+XL_L
B_b_ion
B_y_ion
y10++
y11++
y5+
b5+
y6+
y7+
y8+
y4+
y9++
y2+
b3+
y5++
y7++
b8+
b9+
y9+
y10+
b11+
